# Genomic Sequencing and Comparative Analysis of Epstein-Barr Virus Genome Isolated from Primary Nasopharyngeal Carcinoma Biopsy

**DOI:** 10.1371/journal.pone.0036939

**Published:** 2012-05-10

**Authors:** Hin Kwok, Amy H. Y. Tong, Chi Ho Lin, Si Lok, Paul J. Farrell, Dora L. W. Kwong, Alan K. S. Chiang

**Affiliations:** 1 Department of Paediatrics and Adolescent Medicine, Li Ka Shing Faculty of Medicine, The University of Hong Kong, Hong Kong, China; 2 Genome Research Centre, Li Ka Shing Faculty of Medicine, The University of Hong Kong, Hong Kong, China; 3 Department of Clinical Oncology, Li Ka Shing Faculty of Medicine, The University of Hong Kong, Hong Kong, China; 4 Section of Virology, Imperial College Faculty of Medicine, London, United Kingdom; The Chinese University of Hong Kong, Hong Kong

## Abstract

Whether certain Epstein-Barr virus (EBV) strains are associated with pathogenesis of nasopharyngeal carcinoma (NPC) is still an unresolved question. In the present study, EBV genome contained in a primary NPC tumor biopsy was amplified by Polymerase Chain Reaction (PCR), and sequenced using next-generation (Illumina) and conventional dideoxy-DNA sequencing. The EBV genome, designated HKNPC1 (Genbank accession number JQ009376) is a type 1 EBV of approximately 171.5 kb. The virus appears to be a uniform strain in line with accepted monoclonal nature of EBV in NPC but is heterogeneous at 172 nucleotide positions. Phylogenetic analysis with the four published EBV strains, B95-8, AG876, GD1, and GD2, indicated HKNPC1 was more closely related to the Chinese NPC patient-derived strains, GD1 and GD2. HKNPC1 contains 1,589 single nucleotide variations (SNVs) and 132 insertions or deletions (indels) in comparison to the reference EBV sequence (accession number NC007605). When compared to AG876, a strain derived from Ghanaian Burkitt's lymphoma, we found 322 SNVs, of which 76 were non-synonymous SNVs and were shared amongst the Chinese GD1, GD2 and HKNPC1 isolates. We observed 88 non-synonymous SNVs shared only by HKNPC1 and GD2, the only other NPC tumor-derived strain reported thus far. Non-synonymous SNVs were mainly found in the latent, tegument and glycoprotein genes. The same point mutations were found in glycoprotein (*BLLF1* and *BALF4*) genes of GD1, GD2 and HKNPC1 strains and might affect cell type specific binding. Variations in LMP1 and EBNA3B epitopes and mutations in *Cp* (11404 C>T) and *Qp* (50134 G>C) found in GD1, GD2 and HKNPC1 could potentially affect CD8^+^ T cell recognition and latent gene expression pattern in NPC, respectively. In conclusion, we showed that whole genome sequencing of EBV in NPC may facilitate discovery of previously unknown variations of pathogenic significance.

## Introduction

Epstein-Barr virus (EBV) is a ubiquitous human gammaherpesvirus infecting more than 90% of the world's population and is associated with both non-malignant disease, such as infectious mononucleosis, as well as malignant diseases, such as nasopharyngeal carcinoma (NPC), endemic Burkitt's lymphoma, Hodgkin's disease, B- and T-cell lymphomas and rare cases of gastric carcinoma [1]. The EBV genome comprises approximately 170 kb and contains at least 86 open reading frames. The virus genome contains a long unique region interspersed by four major internal repeats (IR1 to IR4) and terminal repeats (TR). Nine latent proteins including Epstein-Barr nuclear antigen 1 (EBNA1), EBNA2, EBNA3A, -3B, -3C, EBNA-LP and latent membrane protein 1 (LMP1) and LMP2A, -2B are encoded by genes situated in the unique region of the genome [1]. Other open reading frames encode capsid proteins, transcription factors as well as lytic proteins of various functions [2]. In addition to protein-coding genes, EBV genome also encodes non-coding EBV RNAs, such as Epstein-Barr virus–encoded small RNA 1 (*EBER1*) and 2 (*EBER2*), BART-derived microRNAs (*miRNAs-BARTs*) and BHRF1 microRNAs (*miRNAs-BHRF1*) [3], [4].

Four complete or partial EBV genomes, B95-8, AG876, GD1 and GD2, have been reported. The prototypic EBV strain B95-8 is the first complete genome sequenced and was derived from an individual with infectious mononucleosis [5]. A more representative type 1 EBV reference genome (Genbank accession: NC_007605) was constructed using B95-8 as the backbone while an 11-kb deleted segment was provided by Raji sequences [6]. AG876 originated from a Ghanaian case of Burkitt's lymphoma and is the only complete type 2 EBV sequence available to date [7], [8]. GD1 and GD2 are EBV genomes derived from NPC patients from the Guangdong province of southern China. GD1 was isolated from saliva of a NPC patient [9], while GD2 was isolated from an NPC tumor [10].

The consistent association of undifferentiated NPC with EBV implies that EBV plays a causal role in NPC development. Type 1 & 2 EBV have long been observed to display a characteristic geographical prevalence [11]. Similarly, the incidence of NPC has a remarkable geographical pattern, as it is much more frequent in Southeast Asia, North Africa, and Alaska than in the rest of the world [12]. The concordance of geographical distribution of EBV strains and the endemic incidence of NPC have prompted studies to investigate whether distinct strains of EBV might contribute to disease. EBV strains have previously been characterized in NPC tumors using strain-specific markers in the *EBER1* and *-2*, *LMP1*, *BHRF1*, *BZLF1* and *EBNA1* loci in samples from China, south Asia, and northern Africa [13]–[18]. These genes were chosen either because they are expressed in NPC, or they play an important role in the EBV infectious cycle. *LMP1* deletions and point mutations were suggested to be associated with lymphoproliferative diseases [19]. The frequent association of an *LMP1* deletion variant Asp335 with NPC in Hong Kong was also reported [20]. Similarly, evidence supports a role for selection of a del-*LMP1* over the wt-*LMP1* variants in NK/T-cell lymphoma in the same Hong Kong population [21]; a specific *EBNA1* subtype (V-val), also showed preferential occurrence in NPC biopsies [22]. These observations support the notion of pathogenic strains in NPC. However, functional assays on seven *LMP1* variants failed to show differences *in vitro* transformation assays or in observed signaling properties [23]. Despite these results, the continued predominance of China 1, an *LMP1* variant observed in NPC tumor over other strains found in circulation [24] argued for the selection of contributory strains in tumorigenesis. Genetic variations in the small subsets of genes investigated thus far are not sufficient for unequivocal identification of all but a small number of EBV strains to assess their geographical distribution and precise association to disease. There is an unmet need for further whole genome sequencing analysis of EBV.

GD1 was the EBV genome sequenced from saliva of a NPC patient using PCR amplification and sub-cloning followed by conventional dideoxy-based DNA sequencing. GD2 was the first EBV sequence determined by the so termed next-generation sequencing technology. Using the Illumina (Solexa) platform, the sequence of the GD2 genome was recently obtained from random shotgun sequencing and assembly of total DNA sequences derived from the primary NPC tumor [10]. However, sequencing total cellular & viral DNA in a sample is costly and inefficient due to the relatively small quantity of viral DNA present in the tumor sample, thereby limiting the generation of the high read depth necessary to make high confident base calls of the viral genome. One way in dealing with the problem is to use target enrichment technology to increase the relative amount of viral DNA [25]. Here we reported another approach of PCR enrichment (Amplicon Sequencing) followed by sequencing the amplified products on the Illumina Genome Analyzer IIx platform to determine the genome sequence of an EBV isolate from NPC tumor of a Chinese patient in Hong Kong. The EBV genome sequence was assembled with reference to wild type EBV and designated as HKNPC1 (Genbank accession number JQ009376). Phylogenetic analysis was performed on HKNPC1 and the four EBV strains available in Genbank. We compared the distribution of synonymous and non-synonymous base differences in the four EBV strains and assessed their potential roles in pathogenesis. Although geographic variations may account for many mutations detected, we identify a glycoprotein SNV as potential marker of epithelial viral strains, and mutations in *Cp* and *Qp* latent promoters to which may contribute to latent gene expression pattern in NPC. We also identify variations in EBNA3B epitopes, which may alter CD8^+^ T cell recognition. Future elucidation of additional EBV genomes in NPC tumors and local wild type EBV strains is warranted to provide further insights relating geographical distribution to disease.

## Results

### Summary of sequencing data of HKNPC1 genome

**Figure 1:**
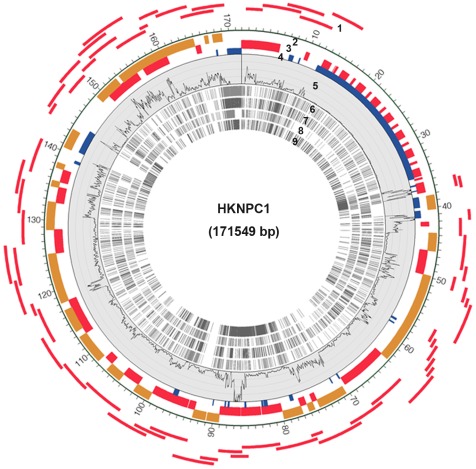
Circular representation of HKNPC1 genome. Numbered tracks represent the following: (1) PCR amplicons prepared for next-generation sequencing (NGS); (2) reverse open reading frames; (3) forward open reading frames; (4) repeat regions; (5) read depth of NGS reads, height of the track represents 10,000 reads; single nucleotide variations (SNVs) and indels of (6) HKNPC1, (7) GD1, (8) GD2, and (9) AG876, in comparison to reference EBV sequence. This figure was created using Circos software [37].

The non-repetitive regions of the HKNPC1 genome, represented by 60 overlapping amplicons, were generated using pairs of PCR primers targeting previously recognized regions of sequence conservation. A total of 3,417,075 sequence reads of 76-base pair-end reads were generated resulting in 260 Mb of sequence data from the combined pool of amplicons. Sequence read that passed default quality control filters on the Illumina platform were aligned to EBV reference genome (accession number NC007605) sequence followed by BLAST procedure against nucleotide database to determine the sequence identity. 91.99% (3,143,389) of the reads can be mapped to the reference genome indicating the high specificity of the amplicon generation. The aligned sequence covered 99.98% of the expected amplified regions of the EBV genome. Only base positions with a read depth of at least 5 reads were used to tabulate the consensus sequence of HKNPC1. Enrichment of EBV sequence by PCR before Illumina DNA sequencing enabled a high average read depth of 1,688-fold across the targeted region with approximately 52% of the PCR-amplified regions of HKNPC1 having greater than 1,000-fold read depth coverage (Table 1, Figure 1). We attempted to validate ambiguous positions with insufficient read depth (less than 5 reads) by dideoxy-based DNA sequencing. However, 153 nucleotide positions remained unvalidated and were marked as N in the genome sequence. One copy of internal repeat 1 (*IR1*) and one copy of terminal repeat (*TR*) were sequenced by conventional dideoxy-based sequencing, whilst the copy numbers of *IR1* and *TR* of reference EBV genome were adopted in our assembly. The gaps in 3′ flanking region of *IR-1*, *IR-2*, and *IR-4* (reference EBV coordinates 35,273–36,132; 38,194–40,615; and 139,950–143,125, respectively) were also represented by tracts of Ns. Accordingly, the present study represents a useful high-resolution draft comprising all the known coding and regulatory sequences but has not resolved all the repetitive regions. The HKNPC1 draft genome is approximately 171,549 bp with GC-content of 59.5%. It was annotated using the information derived from the reference EBV sequence. The high sequence depth allowed us to assess genetic heterogeneity within our isolate. A position was defined to be homogeneous if the variant frequency is > = 95% and a position to be heterogeneous if the variant frequency is between 20% and 94%, both homogenous and heterogeneous positions require read depth to be 5 or above While we could not completely rule out artifactual sequence alterations that might occur during amplicon generation or during Illumina sequencing, we identified a total of 172 potential heterogeneous nucleotide positions in HKNPC, of which 143 were located in repeat sequences throughout the genome. The remaining 29 heterogeneity positions were distributed within the coding sequences of the latent genes *EBNA1*, *-2*, *-3A*, and *LMP1;* the tegument protein genes, *BPLF1*, *BOLF1* and *BGLF1*; other genes such as *BVLF1*, *BFLF2* and *BFRF3*; and within the intergenic regions (Table S1).

**Table 1:** Depth and coverage of reads of HKNPC1.

Read Depth	Mapped data (bp)	Percentage coverage of PCR-amplified regions
> = 1	141,468	99.98%
>10	141,233	99.82%
>100	138,321	97.76%
>500	116,966	82.66%
>1000	74,028	52.32%

### HKNPC1 showed closer phylogenetic relationship to GD1 and GD2 than B95-8 and AG876

**Figure 2:**
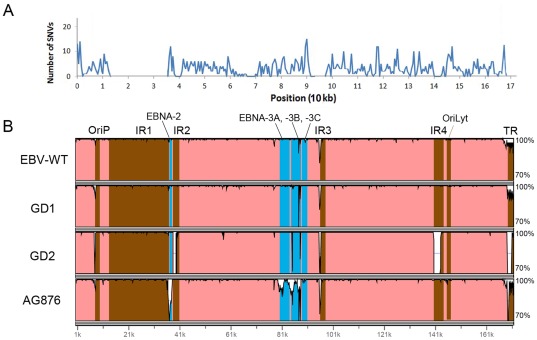
Comparison of HKNPC1 genome to other EBV genomes. (A) Genome-wide distribution of SNVs of HKNPC1. Coordinates of SNVs were extracted by cross_match software and a density plot was constructed using 500-bp non-overlapping windows. (B) Similarity graphs of reference EBV, GD1, GD2 and AG876 compared against HKNPC1. The four genomes generally have high sequence identity with HKNPC1, except repeat regions (brown) and polymorphic genes (blue). *EBNA2*, *EBNA3A*, *-3B*, and *-3C* show lower identity in AG876 (type 2), than the other three type 1 EBV genomes. The figure was generated by mVISTA software, using 100-bp moving window with minimum identity of 70% and maximum identity of 100%.

**Figure 3:**
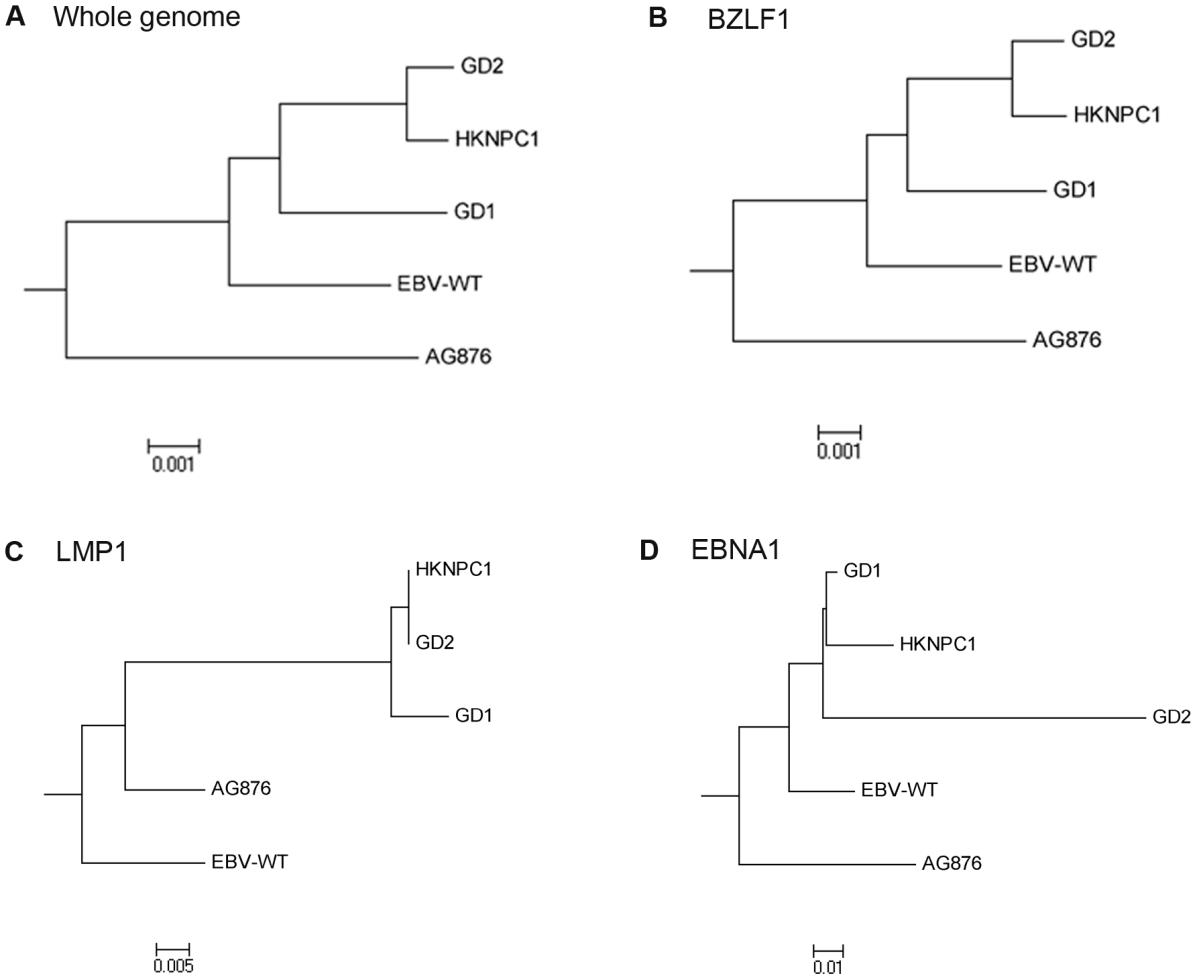
Phylogenetic analysis of the five EBV genomes. Phylogenetic tree based on DNA and protein sequences of the five EBV strains. (A) DNA sequence of complete genome of the five strains, with poorly aligned and highly divergent sequences masked by Gblocks. Phylogenetic trees based on protein sequence alignment of (B) BZLF1, (C) LMP1, and (D) EBNA1 were generated. All these trees showed a closer distance among the three NPC-related EBV strains, GD1, GD2, and HKNPC1, than the other two viral strains. Phylogenetic analysis was performed using MEGA software (version 5), by Neighbor-joining (NJ) algorithm. Divergence scale, in numbers of substitution per site, is shown under each tree.

Single nucleotide variations (SNVs) in the consensus HKNPC1 genome compared to the reported strains were extracted by cross_match (http://www.phrap.org/phredphrapconsed.html). After masking the repeat regions, a high density of SNVs was observed in the previously reported polymorphic *EBNA2*, *EBNA3* and *LMP1* loci (Figure 2A). The region where *BMRF1* resided had low SNV density, with no SNV present from positions 67,659 to 69,061 (HKNPC1 coordinates). Pairwise alignment of HKNPC1 with the four reported strains was performed and visualized by mVISTA (http://genome.lbl.gov/vista/mvista/submit.shtml) (Figure 2B). Multiple whole sequence alignment of HKNPC1 and the other four EBV subtypes were performed using MAFFT [26] employing Gblocks [27] to mask poorly aligned positions and divergent regions of the aligned sequences. Overall sequence similarities between HKNPC1 and the four other strains were high, reaching 98.6% (B95-8), 98.5% (GD1), 95% (GD2) and 96.6% (AG876), respectively. Expectedly, low similarity regions coincided with regions of high SNV density, exemplified in the polymorphic regions spanning *EBNA2* and *EBNA3A*, *-3B* and -*3C*. The three type 1 EBV genomes, B95-8, GD1 and GD2, have higher sequence similarity with HKNPC, particularly in the regions spanning *EBNA2* and *EBNA3*, indicating that HKNPC1 is a type 1 virus. Neighbour-joining trees constructed using software MEGA5 [28] showed that GD1, GD2 and HKNPC1 are more closely related (Figure 3A). Gene trees generated from alignment of translated amino acid sequences of *BZLF1*, *LMP1* and *EBNA1* provided the same result (Figure 3B to 3D). Sequences of these genes were subsequently validated by dideoxy-based sequencing.

### Single nucleotide variations (SNVs) shared by Chinese derived GD1, GD2 and HKNPC1

**Figure 4:**
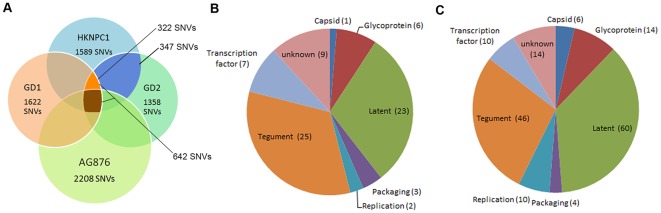
Summary of single nucleotide variations and non-synonymous mutations in GD1, GD2 and HKNPC1 genomes. (A) Number of single nucleotide variants (SNVs) of HKNPC1, GD1, GD2 and AG876 with each genome compared against reference EBV. The orange region represents the 322 SNVs shared by GD1, GD2 and HKNPC1 with exclusion of those of AG876, whereas the blue region represents the 347 SNVs shared by GD2 and HKNPC1 with exclusion of those of AG876 and GD1. (B) Non-synonymous SNVs shared by GD1, GD2 and HKNPC1 were categorized by protein function based on the work of Tabouriech et al. [2]. (C) Non-synonymous SNVs shared by GD1, GD2 and HKNPC1 and those shared by GD2 and HKNPC1 were pooled together and categorized by function of the genes.

HKNPC1 contains 1,589 single nucleotide variations (SNVs) and 132 indels when compared to the reference EBV genome (Accession no. NC007605). Of the 1,589 SNVs, 1,167 had a read depth of 100 or above. The remaining 422 SNVs with read depth less than 100 were verified by dideoxy-DNA sequencing. While 1,043 of the SNVs were found in coding sequence, none were located within the consensus TATA box or PolyA adenylation motifs. The Chinese derived GD1, GD2 and HKNPC1 isolates shared 642 SNVs when compared to the reference genome (Figure 4A). Discounting SNVs found in AG876, which is derived from Ghanaian Burkitt's lymphoma, 332 SNVs remained (orange region in figure 4A) with 76 of these representing non-synonymous nucleotide changes (Table S2 and figure 4). Since GD1 originated from saliva of a NPC patient, GD2 and HKNPC1 represent the only two direct NPC tumor-derived strains reported thus far. By considering SNVs that are shared only between these two tumor-derived strains, additional 347 SNVs were identified (blue region in figure 4A) of which 88 were non-synonymous mutations (Table S2 and figure 4). Of note, the total number of SNVs found in HKNPC1 isolate would be underestimated due to incompleteness of the genome. However, the number of shared SNVs among GD1, GD2 and HKNPC1 strains would still be accurately represented in our comparative analyses since the majority of shared SNVs among viral strains are predominantly located at the non-repeat regions of the genome. In total, we reported 164 non-synonymous SNVs among the three viral strains. A large proportion of them is located in latent (59/164) and tegument (46/164) proteins, followed by glycoproteins (14/164) (figure 4C, Table S2). The remaining SNVs were situated in genes encoding proteins involving in replication, packaging, transcription, capsid structure, or those of unknown function (Table 2).

**Table 2:** EBV genes with non-synonymous single nucleotide variations shared by NPC-derived EBV strains.

Category	Gene	Protein product	No. of SNVs*
Capsid	*BVRF2*	Protease	2
	*BTRF1*	Capsid	2
	*BVRF1*	Portal plug	2
Glycoprotein	*BDLF3*	gp150	4
	*BLLF1*	gp350	4
	*BALF4*	gB	4
	*BKRF2*	gL	2
Latent	*EBNA1*	EBNA1	7
	*EBNA2*	EBNA2	7
	*EBNA3A*	EBNA3A	5
	*EBNA3B*	EBNA3B	8
	*EBNA3C*	EBNA3C	6
	*LMP1*	LMP1	15
	*LMP2A*	LMP2A	12
Packaging	*BGRF1/BDRF1*	Terminase small subunit	1
	*BFLF1*	BFLF1	3
Replication	*BALF5*	Polymerase	1
	*BALF2*	BALF2	3
	*BBLF2/BBLF3*	Primase-associated factor	6
Tegument	*BNRF1*	Major tegument protein	3
	*BRRF2*	unknown function	10
	*BOLF1*	LTP-binding protein	4
	*BPLF1*	Large tegument protein	18
	*BGLF1*	BGLF1	2
	*BKRF4*	BKRF4	3
	*BSLF1*	Primase	5
	*BRLF1*	Rta	1
Transcription factor	*BHRF1*	bcl-2 homolog	1
	*BSLF2/BMLF1*	SM protein	3
	*BBRF1*	portal protein	1
	*BZLF1*	Zta	3
	*BBRF2*	BBRF2	2
unknown	*BFRF2*	unknown function	9
	*BcRF1*	unknown function	3
	*BFRF1A*	unknown function	2

* SNVs, single nucleotide variations.

### Shared SNVs in protein-encoding sequences

**Figure 5:**
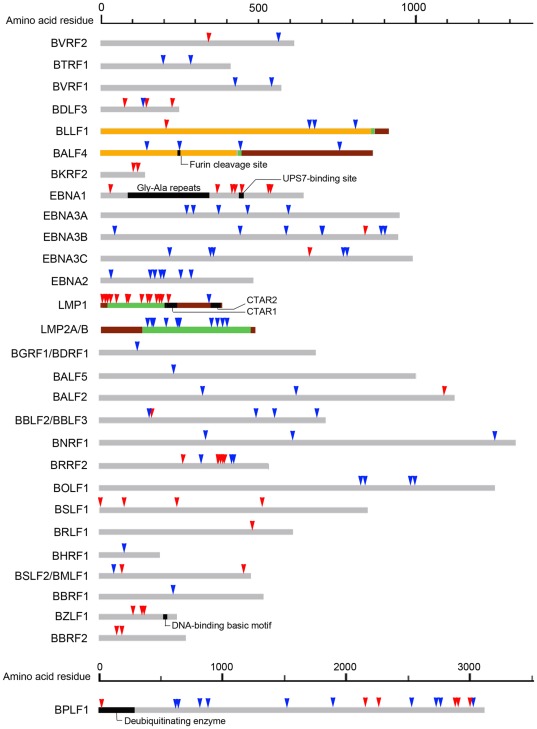
Location of amino acid changes of EBV proteins encoded by GD1, GD2 and HKNPC1 genomes. Amino acids changes in EBV proteins with known or putative function due to non-synonymous SNVs are marked by arrows. Red arrows indicate amino acid changes shared by HKNPC1, GD1 and GD2, but not in AG876. Blue arrows indicate amino acid changes shared by HKNPC1 and GD2, but not in GD1 and AG876. Known or predicted cytoplasmic domain (brown), transmembrane domain (green) and extracellular domain (yellow) of membrane proteins are illustrated. Black bars represent specialized features of BALF4 (glycoprotein B), EBNA1, LMP1, EBNA1 and BPLF1.

Compared to the reference genome, all latent genes of the two tumor-derived EBV strains, GD2 and HKNPC1, harbor non-synonymous SNVs. *LMP1* and *-2* have the highest number of non-synonymous mutations, 14 and 12 SNVs, respectively. All but one such SNV in *LMP1*, and all those in *EBNA1*, were shared amongst GD1, GD2 and HKNPC1. All non-synonymous SNVs in *LMP2*, *EBNA2* and *EBNA3s* except one in *EBNA3C*, were shared by GD2 and HKNPC1. The majority of SNVs in *LMP1*, and all SNVs in *LMP2*, resulted in amino acid changes in transmembrane region of the encoded proteins (Figure 5). Cytoplasmic domains of LMP1 and -2, which carry out important biological function, are more conserved. Based on strain-determining amino acids of EBNA1 (A487V, D499E and T524I) and C-terminus of LMP1 (G212S, Q334R, L338S), HKNPC1 can be classified into the V-val and China 1 genotypes. A 30-bp deletion is also found in *LMP1*, leading to a 10-amino acid deletion in the C-terminus of the encoded protein, as in GD1, GD2 and AG876. Among the non-synonymous SNV-containing tegument loci, *BPLF1*, and *BRRF2*, have the highest number of shared SNVs (18 and 10 SNVs, respectively). The 18 non-synonymous SNVs in the tegument loci are distributed among *BNRF1*, *BGLF1*, *BKRF4*, *BSLF1* and *BRLF1*. The glycoprotein-encoding genes, *BDLF3*, *BLLF1*, *BALF4* and *BKRF2*, which encode gp150, gp350, gB and gL, respectively, contained shared SNVs. Three non-synonymous mutations in *BZLF1* shared by the two NPC-derived strains, were not located in the known DNA-binding region.

### Variations in latent EBV-specific epitopes

Amongst the shared non-synonymous SNVs of the Chinese derived GD1, GD2 and HKNPC1 isolates, 34 are associated in known EBV-specific epitopes (Table S3); 19 and 15 are found in CD8^+^ and CD4^+^ epitopes, respectively. Amino acid changes in CD8^+^ epitopes were identified in all latent proteins, including EBNA1, -2, -3A, -3B, -3C, and LMP1 and -2, while only EBNA1, -2, LMP1 and -2 contain residue changes in CD4^+^ epitopes. Five CD8^+^ epitopes harboring amino acid changes were identified to be restricted through HLA alleles particularly common in the southern Chinese population. AVF and IVT epitopes in EBNA3B and SSC epitope in LMP2 are restricted through HLA 1 A11, whereas the FRR epitope in LMP1 and IED are restricted through HLA 1 B40.

### Variations in non-coding genes and promoter sequences

MicroRNAs in *BHRF1* (*miR-BHRF1*) region and *BART* region (*miR-BART*) and Epstein-Barr virus–encoded small RNAs (*EBERs*) are non-coding genes. Genes encoded for microRNAs *BHRF1* (*miR-BHRF1-1 to -3*) and *EBER1* are highly conserved with no variations among the five strains, whereas in the *miR-BARTs*, only a single point mutation (147,821 T>A) was observed in the sequence encoding *miR-BART19-5p*. Variations were observed in *EBER2*, however, only one point mutation (7,048 A>C) is shared by GD2 and HKNPC1, and other mutations are not exclusive to NPC-derived strains. The *BZLF1* promoter was classified to be variant Zp-V3 based on strain-defining mutations at −196, −141, −106 and −100 from the transcription start site. Although no mutations were identified in known and predicted TATA boxes, sequence changes of 11,324 G>T and 11,404 C>T in *Cp*, and 49,937 G>A and 50,134 G>C in *Qp* were observed. Mutations at 11,324, 11,404 and 44,937 were present in GD1, GD2 and HKNPC1, but not in AG876 and wild type, while mutation at 50,134 was found in GD2 and HKNPC1, but not in the other three strains.

## Discussion

Genotyping studies to date have focused on different regions of EBV, making comparisons across studies difficult, and limiting our ability to define the full spectrum of diversity within the EBV genome. Many studies aimed to identify NPC-specific strains focused only on a limited number of genes expressed in NPC, predominantly *EBNA1*, *LMP1*, *LMP2A*, *BZLF1*, *miR-BART*, and *EBER1* and *-2*. We extended our analysis across other regions of the genome and identified a host of SNVs shared among the three NPC-associated strains. By only considering mutations common in HKNPC1, GD1 and GD2, false positives caused by sequencing errors can be minimized. Since it is known that latent genes are polymorphic, it is not surprisingly that we observed a concentration of non-synonymous SNVs among latent genes in our study. We also observe a significant number of SNVs in the genes encoding the tegument proteins and the glycoproteins.

Studies of EBV's role in NPC pathogenesis were mainly restricted to genes expressed in NPC. The role of other EBV life cycle genes in NPC has not been well studied. One question related to the EBV infectious cycle is whether there is preferential entry of certain EBV strains into epithelial cells. Such a mechanism may involve membrane glycoproteins, which are responsible for cell type specific binding. The GD2 and HKNPC1 strains were of epithelial origin, while B95-8 and AG876 were from Burkitt's lymphoma cell lines. Despite the fact that GD1 was not directly harvested from epithelial tissue of an NPC patient, it is reasonable to assume that GD1 is also an epithelial strain released from lymphoepithelial organ (mucosa-associated lymphoid tissue, e.g., tonsils) into saliva. Non-synonymous mutations in glycoprotein genes *BLLF1* (79,265 C>G) and *BALF4* (157,568 C>T) were found common in HKNPC1, GD1 and GD2. These mutations cause amino acid changes at the CR2 receptor binding site of gp350 (E201Q) and at the furin cleavage site of gB (D433N) proteins, respectively. However, the functional consequences of these mutations are not known. Comparison of the sequences of NPC-derived EBV genomes with those of lymphoid-derived EBV genomes of the same individuals or individuals of the same population may contribute to our understanding of the significance of these mutations. Variants of two HLA A11-restricted immunodominant epitopes in EBNA3B protein were found in HKNPC1. These were AVF epitope with a mutated fourth residue (D>N) and IVT epitope with a mutated ninth residue (K>N) and were reported to be poorly recognized by IVT- and AVF-specific cytotoxic T cells compared to the wild-type epitopes [29]. Of interest, computer-based analysis suggested these non-immunogenic variant epitopes were under positive selection by the immune system [30]. A substitution of Leu to Phe was found in the second residue of an HLA A2-restricted epitope YLL of LMP1 protein. This substitution was found to be more prevalent in viral strains contained in NPC specimens than those in adjacent non-neoplastic nasopharyngeal tissue of southern Chinese and Taiwanese patients [31]. The EBNA3B and LMP1 variant epitopes might contribute to evasion from T-cell surveillance. HKNPC1, GD1 and GD2 strains shared the *Cp* mutation (11404 C>T), whereas only GD2 and HKNPC1 shared the *Qp* mutation (50134 G>C). It was reported that the mutation in *Cp* reduced its promoter activity while that in *Qp* increased its activity [32]. Furthermore, both mutations were found to be more prevalent in EBV strains harbored in NPC tumors than in peripheral blood mononuclear cells [32].

We observed a number of heterogeneous nucleotide positions in HKNPC1. The majority of these positions were found in different repeat regions of the EBV genome. These heterogeneous nucleotide positions could be caused by polymerase slippage in sequencing or arise during amplicon generation. With our high sequencing depth, it is also possible that some of the observed sequence heterogeneity was contributed by low numbers of infiltrating lymphocytes in undifferentiated NPC or by actual heterogeneity of the viral population within the tumor. Nevertheless, low number of heterogeneous positions observed is consistent with the current view of monoclonal origin of EBV in NPC The possibility of a low level of spontaneous mutations occurring during the course of clonal expansion should be further investigated.

A recent study reported the characterization of an EBV genome contained in a NPC biopsy, designated GD2, by direct shotgun sequencing of the total tumor DNA. However, the yield of reads of the viral genome was very low, comprising less than 0.014% of the total sequence reads, from an average of no more than six copies of EBV per tumor cell [10]. In contrast, the present amplicon-based sequencing approach yielded mappable reads of more than 90%, thereby greatly increasing the efficiency and economy of next-generation sequencing technology. We envisage that direct EBV sequencing would be difficult for non-tumor or mixed tumor sample with low viral load without the use of an enrichment step. In addition to amplicon sequencing, it should be feasible to apply sequence capturing technologies used in human exome sequencing to EBV sequence enrichment as exemplified by the recent study of specific capture and whole-genome sequencing of herpesvirus genome from clinical samples [25]. These and other approaches might further reduce cost and time in whole-genome sequencing to enable larger survey of a much greater number of EBV isolates.

In summary, we have reported the sequence of an EBV genome isolated from a primary NPC tumor, designated HKNPC1, through amplicon sequencing on the Illumina platform. Comparative analysis reveals variations not only in genes expressed in NPC but also in genes encoding for tegument proteins, glycoproteins and other proteins. A number of single nucleotide variants with potential contribution to NPC pathogenesis is found in the HKNPC1 genome. The results showed the importance of developing a high throughput sequencing approach for direct determination of hundreds of EBV genome sequences to investigate the epidemiological and pathogenic roles of EBV strain variation in EBV-associated diseases.

## Materials and Methods

### Ethics Statement

The NPC tumor was biopsied after obtaining written consent from a 20-year-old Chinese male patient diagnosed in 2008 with nasopharyngeal carcinoma of stage T3N3aM1 prior to treatment at Queen Mary Hospital, Hong Kong, China. Collection of NPC biopsies was approved by Institutional Review Board of The University of Hong Kong/Hospital Authority Hong Kong West Cluster for the purpose of EBV genome sequencing in NPC tumors.

### NPC tumor specimen

Primary tumor material was biopsied by Prof. Dora LW Kwong. Fresh NPC tumor biopsy was temporarily stored in PBS with 1% fetal bovine serum, and DNA extraction was performed within one hour after incision, by Qiagen Blood and Tissue Kit according to the manufacturer's protocol (Qiagen, Hilden, Germany).

### PCR amplication of EBV fragments

Overlapping amplicons representing the non-repetitive regions of the EBV genome were generated using 60 sets of primers (Table S4) and HotstarTaq Plus Kit (Qiagen). 100 ng of tumor DNA was performed using 50 µl reaction mixture, containing 3 µl each of 10 µM forward and reverse primers, 1 µl of 10 mM deoxynucleotide triphosphate (dNTPs), 0.2 µl HotstarTaq Plus enzyme, 5 µl buffer and 10 µl Q solution provided by the kit. Thirty to forty cycles of denaturation (94°C for 45 s), annealing (56°C for 45 s) and extension (72°C for 2 to 6 min) were carried out in automated thermal cycler, where cycle number and extension time depend on length of product. The primer sequences are shown in Table S4 in the supplementary material. Internal repeats and terminal repeats were excluded in PCR. The products were purified by QIAquick PCR purification kit (Qiagen) and QIAEX II gel extraction kit (Qiagen), and were normalized to equal molecular quantity before combining for DNA sequencing.

### Sequence analysis and construction of the HKNPC1 genome

Purified pooled PCR products were fragmented randomly by nebulization. DNA fragments were end-repaired using DNA Terminator End Repair kit (Lucigen, WI, USA), and purified using the QIAquick PCR Purification kit (Qiagen), according to company's protocol. 3′ dA-tailing was carried out by Klenow Fragment (Enzymatics, MA, USA). The dA-tailed DNA fragments were separated and purified on 8% polyacylamide gels (Life Technologies, CA, USA), and a gel slice of insert size ∼100–125 bp was excised and purified using QIAquick Gel Extraction kit (Qiagen). Illumina (CA, USA) Solexa paired-end adaptors were ligated to the purified DNA fragments by Rapid T4 DNA Ligase (Enzymatics). The fragments were amplified for 12 cycles with AccuPrime Pfx DNA Polymerase (Life Technologies). A final size selection of the amplified library was performed using a 2% Low Range Ultra agarose gel (BioRad, CA, USA). The Illumina library was extracted in TE using MinElute Gel Extraction kit (Qiagen). Finally, the library concentration was measured on NanoDrop spectrophotometer (Thermo Scientific, DE, USA) and quantified by quantitative PCR. Sequencing run of 76-base paired-end was performed as manufacturer's recommendations [33]. We carried out quality assessment on the raw reads using Illumina's default parameters to remove reads that were of low quality or comprise adaptor sequences or homopolymer sequences. The high quality reads were aligned to the reference EBV genome (NC_007605) using Burrows-Wheeler Aligner (BWA) version 0.5.8c [34]. Nucleotide variations were identified by using SAMTools [35] with VarScan version 2.2 [36] and by in-house scripts. We defined a position to be homogeneous if the variant frequency is > = 95% and a position to be heterogeneous if the variant frequency is between 20% and 94% (both read depth of 5 or above). Nucleotide positions with read depth less than 5 were classified as ambiguous sites as there is insufficient depth to make a high confidence call.

### Sequence validation

Regions where ambiguous sites are clustered were verified by PCR amplification with specific primers (Table S5) followed by subsequent conventional dideoxy-DNA sequencing. 100 ng of genomic DNA (100 ng in total) was added to a 25 µl reaction mixture, containing 1.5 µl each of 10 µM forward and reverse primers, 0.5 µl of 10 mM deoxynucleotide triphosphate (dNTPs), 0.1 HotstarTaq enzyme, 2.5 µl buffer and 5 µl Q solution provided by the kit. Forty cycles of denaturation (94°C for 45 s), annealing (56°C for 45 s) and extension (72°C for 1 min) were carried out in automated thermal cycler. The products were purified by QIAEX II gel extraction kit (Qiagen).

### Phylogenetic analysis

A global comparison and visualization of HKNPC1 against EBV-WT, GD1, GD2 and AG876 was performed by mVISTA (http://genome.lbl.gov/vista/mvista/submit.shtml), using 100-bp moving window. Phylogenetic analysis was performed using Molecular Evolutionary Genetics Analysis (MEGA) software, version 5.0 [28], by Neighbor-joining (NJ) algorithm, based on multiple sequence alignments of the five whole genomes and individual genes using MAFFT [26]. Poorly aligned sequences were masked by Gblocks [27] before construction of phylogenetic trees.

### Comparative analysis of NPC-EBV genome with prototype genomes

Lists of SNVs and indels were generated for each pairwise comparison of the HKNPC1, GD1, GD2 and AG876 against NC_007605 using cross_match software (http://www.phrap.org/phredphrapconsed.html). SNVs in HKNPC1 with read depth of 100 or above were considered to be accurate. Dideoxy-DNA sequencing was performed to verify those with read depth less than 100. These lists were compared against one another and SNVs which were found to be common in GD1, GD2 and HKNPC1 were sorted out. Subsequently, SNVs also found in AG876 were removed from the list. Similarly, a list of SNVs shared only by GD2 and HKNPC1 were generated by subtracting the common SNVs of GD2 and HKNPC1 from those found in GD1 and AG876. Non-synonymous mutations were tabulated from these sorted lists of SNVs and were categorized by known and putative function of proteins where these non-synonymous mutations were located. Shared SNVs located in known epitopes in coding sequences, and non-coding features, including TATA boxes, polyA signals, microRNAs, and other non-coding RNAs, were also examined.

#### Nucleotide sequence accession number

The full-length sequence of HKNPC1 was submitted to the Genbank database and assigned accession number JQ009376.

## Supporting Information

Table S1 Heterogeneous sites excluding repeat regions.
(DOCX)

Table S2 Non-synonymous mutations and amino acid changes common to GD1, GD2 and HKNPC1.
(DOCX)

Table S3 Variation in CD4+ and CD8+ specific epitopes in EBV latent proteins.
(DOCX)

Table S4 Primers for EBV enrichment in next-generation sequencing.
(DOCX)

Table S5 Primers for validation by Sanger sequencing.
(DOCX)
