# Supplementary material for: Genomic Sequencing and Comparative Analysis of Epstein-Barr Virus Genome Isolated from Primary Nasopharyngeal Carcinoma Biopsy
Source: PLoS One. 2012 May 10;7(5):e36939. doi: 10.1371/journal.pone.0036939 (PMC3349645; doi:10.1371/journal.pone.0036939)
Supplement: Table S1 — Heterogeneous sites excluding repeat regions. (DOCX) [file pone.0036939.s001.docx]

**Table S1. Heterogeneous sites excluding repeat regions.**

| **Position*** | **Reference** | **Variants** | **# Reference Reads** | **# Variants Reads** | **Frequency** | **Depth** | **Gene** |
| --- | --- | --- | --- | --- | --- | --- | --- |
| 7125 | T | G | 239 | 719 | 75.05 | 958 | intergenic |
| 7189 | A | G/T | 42 | 1\|69 | 0.89\|61.61 | 112 | intergenic |
| 7191 | T | A/C | 70 | 40\|1 | 36.04\|0.90 | 111 | intergenic |
| 8613 | G | T | 382 | 97 | 20.25 | 479 | intergenic |
| 36289 | A | C/G/T | 1999 | 549\|15\|3 | 21.40\|0.58\|0.12 | 2566 | *EBNA2* |
| 36818 | C | A/G/T | 673 | 1\|1\|5696 | 0.02\|0.02\|89.41 | 6371 | *EBNA2* |
| 37672 | T | A/C/G | 461 | 2\|4794\|2 | 0.04\|90.71\|0.04 | 5285 | *EBNA2* |
| 43807 | C | A/T | 33 | 1\|593 | 0.16\|94.58 | 627 | *BFLF2* |
| 49353 | A | G/T | 255 | 3172\|2 | 92.48\|0.06 | 3430 | *BFRF3* |
| 51061 | C | T | 136 | 57 | 29.53 | 193 | *BPLF1* |
| 51075 | C | A/G | 0 | 1\|9 | 10.00\|90.00 | 10 | *BPLF1* |
| 51076 | A | T | 11 | 10 | 32.26 | 31 | *BPLF1* |
| 51824 | T | C | 218 | 1687 | 88.32 | 1910 | *BPLF1* |
| 52226 | G | A/C | 222 | 733\|1 | 76.51\|0.10 | 958 | *BPLF1* |
| 52244 | T | C | 80 | 643 | 88.93 | 723 | *BPLF1* |
| 58244 | C | T | 84 | 149 | 63.95 | 233 | *BPLF1* |
| 58675 | A | C/G/T | 433 | 1\|138\|2 | 0.17\|23.35\|0.34 | 591 | *BPLF1* |
| 59509 | T | A/C | 2575 | 2\|807 | 0.05\|20.99 | 3844 | *BOLF1* |
| 62169 | A | G | 168 | 127 | 31.91 | 398 | *BOLF1* |
| 81095 | A | C/G | 92 | 2\|985 | 0.19\|91.29 | 1079 | *EBNA3A* |
| 96684 | T | C | 24 | 310 | 92.81 | 334 | *EBNA1* |
| 108360 | A | C | 383 | 121 | 24.01 | 504 | intergenic |
| 108361 | T | A/G | 397 | 1\|113 | 0.20\|22.11 | 511 | intergenic |
| 114866 | A | G | 124 | 36 | 21.05 | 171 | *BGLF1* |
| 114867 | G | A | 94 | 62 | 39.49 | 157 | *BGLF1* |
| 134887 | C | G/T | 121 | 900\|1 | 88.06\|0.10 | 1022 | *BVLF1* |
| 147069 | C | T | 165 | 275 | 62.22 | 442 | intergenic |
| 152975 | C | A/G/T | 13 | 43\|2\|356 | 10.26\|0.48\|84.96 | 419 | intergenic |
| 157598 | G | A/C | 25 | 471\|2 | 94.58\|0.40 | 498 | *A73* |
| 167811 | C | T | 2 | 11 | 84.62 | 13 | *LMP1* |

*Coordinates of NC007605
