# Supplementary material for: Genomic Sequencing and Comparative Analysis of Epstein-Barr Virus Genome Isolated from Primary Nasopharyngeal Carcinoma Biopsy
Source: PLoS One. 2012 May 10;7(5):e36939. doi: 10.1371/journal.pone.0036939 (PMC3349645; doi:10.1371/journal.pone.0036939)
Supplement: Table S2 — Non-synonymous mutations and amino acid changes common to GD1, GD2 and HKNPC1. (DOCX) [file pone.0036939.s002.docx]

**Table S2. Non-synonymous mutations and amino acid changes common to GD1, GD2 and HKNPC1.**

| **EBV Coordinate*** | **Nucleotide change** | **Gene** | **Residue** | **B95-8** | **AG876** | **GD1** | **GD2** | **HKNPC1** |
| --- | --- | --- | --- | --- | --- | --- | --- | --- |
| 159 | C/G | *LMP2A* | 153 | T | T | T | **S** | **S** |
| 207 | G/A | *LMP2A* | 169 | S | S | S | **N** | **N** |
| 213 | A/C | *LMP2A* | 171 | Y | Y | Y | **S** | **S** |
| 410 | C/A | *LMP2A* | 208 | L | L | L | **I** | **I** |
| 613 | C/G | *LMP2A* | 248 | I | I | I | **M** | **M** |
| 629 | G/C | *LMP2A* | 254 | V | V | V | **L** | **L** |
| 632 | C/G | *LMP2A* | 255 | L | L | L | **V** | **V** |
| 1073 | A/G | *LMP2A* | 350 | I | I | I | **V** | **V** |
| 1075 | C/T | *LMP2A* | 350 | I | I | I | **V** | **V** |
| 1134 | T/C | *LMP2A* | 370 | I | I | I | **T** | **T** |
| 1196 | A/C | *LMP2A* | 391 | N | N | N | **H** | **H** |
| 1323 | C/T | *LMP2A* | 406 | C | C | C | **S** | **S** |
| 2711 | G/T | *BNRF1* | 326 | A | A | A | **S** | **S** |
| 3821 | G/A | *BNRF1* | 696 | G | G | G | **R** | **R** |
| 5399 | G/A | *BNRF1* | 1222 | V | V | V | **I** | **I** |
| 36283 | T/G | *EBNA2* | 23 | L | L | L | **R** | **R** |
| 36668 | T/G | *EBNA2* | 151 | I | I | I | **M** | **M** |
| 36702 | A/G | *EBNA2* | 163 | R | R | R | **V** | **V** |
| 36769 | A/G | *EBNA2* | 185 | Q | Q | Q | **R** | **R** |
| 36799 | T/C | *EBNA2* | 195 | M | M | M | **T** | **T** |
| 36951 | C/A | *EBNA2* | 246 | R | R | R | **S** | **S** |
| 37054 | C/A | *EBNA2* | 280 | T | T | T | **N** | **N** |
| 42322 | G/T | *BHRF1* | 79 | V | V | V | **L** | **L** |
| 45489 | A/T | *BFLF1* | 250 | L | L | **Q** | **Q** | **Q** |
| 46106 | G/C | *BFLF1* | 44 | D | D | **E** | **E** | **E** |
| 46134 | A/G | *BFLF1* | 35 | I | I | **T** | **T** | **T** |
| 46368 | C/T | *BFRF1A* | 45 | L | L | **F** | **F** | **F** |
| 46410 | C/A | *BFRF1A* | 59 | L | L | **I** | **I** | **I** |
| 47568 | T/G | *BFRF2* | 17 | C | C | **G** | **G** | **G** |
| 47620 | A/C | *BFRF2* | 34 | Q | Q | **P** | **P** | **P** |
| 47638 | G/A | *BFRF2* | 40 | E | E | **G** | **G** | **G** |
| 47868 | G/A | *BFRF2* | 117 | A | A | A | **T** | **T** |
| 47905 | T/C | *BFRF2* | 129 | V | V | **A** | **A** | **A** |
| 47907 | G/T | *BFRF2* | 130 | V | V | **F** | **F** | **F** |
| 48609 | C/T | *BFRF2* | 364 | R | R | **W** | **W** | **W** |
| 48634 | A/T | *BFRF2* | 372 | H | H | H | **L** | **L** |
| 48904 | G/A | *BFRF2* | 462 | R | R | **Q** | **Q** | **Q** |
| 50134 | G/C | *BPLF1* | 3036 | Q | Q | Q | **E** | **E** |
| 50241 | C/T | *BPLF1* | 3000 | R | R | **Q** | **Q** | **Q** |
| 50557 | G/T | *BPLF1* | 2895 | R | R | **S** | **S** | **S** |
| 50558 | C/T | *BPLF1* | 2895 | R | R | **S** | **S** | **S** |
| 50635 | A/G | *BPLF1* | 2869 | F | F | **L** | **L** | **L** |
| 50947 | G/A | *BPLF1* | 2765 | P | P | P | **S** | **S** |
| 51152 | G/C | *BPLF1* | 2696 | R | R | R | **S** | **S** |
| 51664 | C/T | *BPLF1* | 2526 | D | D | D | **N** | **N** |
| 51665 | C/T | *BPLF1* | 2526 | D | D | D | **N** | **N** |
| 52504 | T/C | *BPLF1* | 2246 | N | N | **D** | **D** | **D** |
| 52709 | C/T | *BPLF1* | 2177 | M | M | **I** | **I** | **I** |
| 53605 | C/A | *BPLF1* | 1879 | V | V | V | **F** | **F** |
| 54705 | C/T | *BPLF1* | 1512 | R | R | R | **Q** | **Q** |
| 56855 | C/A | *BPLF1* | 796 | S | S | S | **A** | **A** |
| 57280 | A/C | *BPLF1* | 654 | S | S | S | **A** | **A** |
| 57296 | A/T | *BPLF1* | 848 | D | D | D | **E** | **E** |
| 57412 | G/T | *BPLF1* | 610 | L | L | L | **I** | **I** |
| 59206 | T/G | *BPLF1* | 12 | T | T | **P** | **P** | **P** |
| 59966 | T/G | *BOLF1* | 996 | S | S | S | **R** | **R** |
| 60028 | G/A | *BOLF1* | 975 | P | P | P | **L** | **L** |
| 60454 | G/T | *BOLF1* | 833 | S | S | S | **Y** | **Y** |
| 60527 | T/C | *BOLF1* | 809 | R | R | R | **G** | **G** |
| 70501 | C/G | *BSLF2/BMLF1* | 465 | G | G | **A** | **A** | **A** |
| 71677 | C/T | *BSLF2/BMLF1* | 73 | R | R | **Q** | **Q** | **Q** |
| 71766 | C/G | *BSLF2/BMLF1* | 43 | E | E | E | **D** | **D** |
| 73063 | C/G | *BSLF1* | 511 | A | A | **P** | **P** | **P** |
| 73064 | C/G | *BSLF1* | 511 | A | A | **P** | **P** | **P** |
| 73861 | C/T | *BSLF1* | 245 | A | A | **T** | **T** | **T** |
| 74364 | T/C | *BSLF1* | 77 | H | H | **R** | **R** | **R** |
| 74587 | C/T | *BSLF1* | 3 | A | A | **T** | **T** | **T** |
| 77452 | C/T | *BLLF1* | 805 | G | G | G | **D** | **D** |
| 77896 | A/C | *BLLF1* | 657 | I | I | I | **R** | **R** |
| 77920 | G/T | *BLLF1* | 649 | A | A | A | **D** | **D** |
| 79265 | C/G | *BLLF1* | 201 | E | E | **Q** | **Q** | **Q** |
| 80841 | G/A | *EBNA3A* | 267 | V | V | V | **I** | **I** |
| 80920 | G/A | *EBNA3A* | 293 | S | S | S | **N** | **N** |
| 81111 | A/G | *EBNA3A* | 357 | T | T | T | **A** | **A** |
| 81417 | C/A | *EBNA3A* | 459 | P | P | P | **T** | **T** |
| 81723 | A/T | *EBNA3A* | 561 | I | I | I | **F** | **F** |
| 83171 | G/A | *EBNA3B* | 36 | G | G | G | **E** | **E** |
| 84414 | G/T | *EBNA3B* | 424 | K | K | K | **N** | **N** |
| 84884 | C/A | *EBNA3B* | 581 | T | T | T | **N** | **N** |
| 85225 | C/A | *EBNA3B* | 695 | Q | Q | Q | **K** | **K** |
| 85229 | C/T | *EBNA3B* | 696 | T | T | T | **I** | **I** |
| 85586 | A/G | *EBNA3B* | 815 | Q | Q | **R** | **R** | **R** |
| 85802 | C/G | *EBNA3B* | 887 | A | A | A | **G** | **G** |
| 85841 | A/G | *EBNA3B* | 900 | Q | Q | Q | **R** | **R** |
| 86795 | A/C | *EBNA3C* | 213 | Q | Q | Q | **H** | **H** |
| 87164 | A/C | *EBNA3C* | 336 | E | E | E | **D** | **D** |
| 87198 | A/C | *EBNA3C* | 348 | I | I | I | **L** | **L** |
| 88122 | C/G | *EBNA3C* | 656 | R | R | **G** | **G** | **G** |
| 88465 | A/G | *EBNA3C* | 769 | Q | Q | Q | **R** | **R** |
| 88501 | A/C | *EBNA3C* | 782 | H | H | H | **P** | **P** |
| 90455 | C/T | *BZLF1* | 138 | G | G | **E** | **E** | **E** |
| 90478 | A/C | *BZLF1* | 130 | F | F | **L** | **L** | **L** |
| 90554 | T/A | *BZLF1* | 105 | Q | Q | **L** | **L** | **L** |
| 91431 | G/T | *BRLF1* | 489 | Q | R | **K** | **K** | **K** |
| 94794 | G/A | *BRRF2* | 261 | D | D | **N** | **N** | **N** |
| 94957 | T/G | *BRRF2* | 315 | V | V | V | **G** | **G** |
| 95133 | A/G | *BRRF2* | 374 | T | T | **A** | **A** | **A** |
| 95141 | A/C | *BRRF2* | 377 | G | G | **R** | **R** | **R** |
| 95142 | G/C | *BRRF2* | 377 | G | G | **R** | **R** | **R** |
| 95167 | T/C | *BRRF2* | 385 | M | M | **T** | **T** | **T** |
| 95187 | A/C | *BRRF2* | 392 | K | K | **Q** | **Q** | **Q** |
| 95197 | A/G | *BRRF2* | 395 | E | E | **G** | **G** | **G** |
| 95265 | T/C | *BRRF2* | 418 | C | C | C | **R** | **R** |
| 95292 | G/T | *BRRF2* | 427 | A | A | A | **S** | **S** |
| 95719 | A/T | *EBNA1* | 20 | T | T | **S** | **S** | **S** |
| 96751 | G/A | *EBNA1* | 363 | G | G | **R** | **R** | **R** |
| 96894 | A/T | *EBNA1* | 411 | E | E | **A** | **A** | **A** |
| 96914 | A/T | *EBNA1* | 418 | H | H | **L** | **L** | **L** |
| 96976 | G/A | *EBNA1* | 439 | A | A | **T** | **T** | **T** |
| 97243 | A/G | *EBNA1* | 528 | I | I | **V** | **V** | **V** |
| 97258 | C/A | *EBNA1* | 533 | L | L | **I** | **I** | **I** |
| 97973 | G/A | *BKRF2* | 102 | G | G | **S** | **S** | **S** |
| 98006 | A/T | *BKRF2* | 113 | T | T | **S** | **S** | **S** |
| 99330 | A/C | *BKRF4* | 162 | Q | Q | **P** | **P** | **P** |
| 99351 | G/T | *BKRF4* | 169 | G | G | **V** | **V** | **V** |
| 99356 | C/A | *BKRF4* | 171 | H | H | **N** | **N** | **N** |
| 102600 | T/G | *BBRF1* | 229 | C | C | C | **G** | **G** |
| 103825 | A/G | *BBRF2* | 56 | I | I | **V** | **V** | **V** |
| 103885 | G/A | *BBRF2* | 76 | V | V | **I** | **I** | **I** |
| 104550 | A/C | *BBLF2/BBLF3* | 691 | I | I | I | **M** | **M** |
| 104555 | T/C | *BBLF2/BBLF3* | 690 | T | T | T | **A** | **A** |
| 104980 | C/G | *BBLF2/BBLF3* | 548 | G | G | G | **A** | **A** |
| 105265 | G/T | *BBLF2/BBLF3* | 496 | P | P | P | **T** | **T** |
| 106273 | T/C | *BBLF2/BBLF3* | 160 | M | M | **V** | **V** | **V** |
| 106293 | A/C | *BBLF2/BBLF3* | 153 | V | V | V | **G** | **G** |
| 112981 | A/T | *BGRF1/BDRF1* | 111 | Q | Q | Q | **L** | **L** |
| 115296 | A/G | *BGLF1* | 264 | L | L | **P** | **P** | **P** |
| 115411 | A/G | *BGLF1* | 226 | S | S | **P** | **P** | **P** |
| 118154 | C/T | *BDLF3* | 209 | A | A | **T** | **T** | **T** |
| 118373 | T/C | *BDLF3* | 136 | T | T | **A** | **A** | **A** |
| 118433 | C/A | *BDLF3* | 116 | A | A | A | **S** | **S** |
| 118562 | T/G | *BDLF3* | 73 | I | I | **L** | **L** | **L** |
| 125272 | C/T | *BcRF1* | 33 | T | T | T | **A** | **A** |
| 126191 | G/A | *BcRF1* | 339 | G | G | G | **R** | **R** |
| 127409 | T/C | *BcRF1* | 745 | C | C | C | **R** | **R** |
| 127992 | G/A | *BTRF1* | 193 | A | A | A | **T** | **T** |
| 128269 | C/A | *BTRF1* | 285 | P | P | P | **Q** | **Q** |
| 134372 | A/G | *BVRF1* | 416 | M | M | M | **V** | **V** |
| 134730 | T/C | *BVRF1* | 535 | M | M | M | **T** | **T** |
| 136646 | G/T | *BVRF2* | 337 | A | A | **S** | **S** | **S** |
| 137316 | A/C | *BVRF2* | 559 | H | H | H | **P** | **P** |
| 155609 | C/T | *BALF5* | 227 | R | R | R | **Q** | **Q** |
| 156637 | G/A | *BALF4* | 743 | A | A | A | **V** | **V** |
| 157568 | C/T | *BALF4* | 433 | D | D | D | **N** | **N** |
| 158143 | T/G | *BALF4* | 241 | K | K | K | **T** | **T** |
| 158481 | G/C | *BALF4* | 128 | D | D | D | **E** | **E** |
| 161036 | T/C | *BALF2* | 1093 | S | S | **G** | **G** | **G** |
| 162476 | T/C | *BALF2* | 613 | I | I | I | **V** | **V** |
| 163364 | C/T | *BALF2* | 317 | V | V | V | **M** | **M** |
| 167859 | C/T | *LMP1* | 335 | G | G | G | **D** | **D** |
| 168229 | C/T | *LMP1* | 212 | G | G | **S** | **S** | **S** |
| 168288 | C/G | *LMP1* | 192 | S | S | **T** | **T** | **T** |
| 168297 | T/G | *LMP1* | 189 | Q | Q | **P** | **P** | **P** |
| 168331 | G/T | *LMP1* | 178 | L | L | **M** | **M** | **M** |
| 168414 | T/G | *LMP1* | 150 | D | D | **A** | **A** | **A** |
| 168433 | A/T | *LMP1* | 144 | F | F | **I** | **I** | **I** |
| 168485 | T/G | *LMP1* | 126 | F | F | **L** | **L** | **L** |
| 168767 | A/C | *LMP1* | 84 | C | C | **G** | **G** | **G** |
| 168772 | G/C | *LMP1* | 82 | A | A | **G** | **G** | **G** |
| 168881 | C/T | *LMP1* | 46 | D | D | **N** | **N** | **N** |
| 168944 | G/T | *LMP1* | 25 | L | L | **I** | **I** | **I** |
| 168967 | C/A | *LMP1* | 17 | R | R | **L** | **L** | **L** |
| 168979 | C/G | *LMP1* | 13 | R | R | **P** | **P** | **P** |
| 169009 | T/C | *LMP1* | 3 | H | H | **R** | **R** | **R** |

*Coordinates of NC007605

#Bold letters represent shared amino acid changes among NPC-derived strains
