# Supplementary material for: Genomic Sequencing and Comparative Analysis of Epstein-Barr Virus Genome Isolated from Primary Nasopharyngeal Carcinoma Biopsy
Source: PLoS One. 2012 May 10;7(5):e36939. doi: 10.1371/journal.pone.0036939 (PMC3349645; doi:10.1371/journal.pone.0036939)
Supplement: Table S3 — Variation in CD4+ and CD8+ specific epitopes in EBV latent proteins. (DOCX) [file pone.0036939.s003.docx]

**Table S3. Variation in CD4+ and CD8+ specific epitopes in EBV latent proteins.**

| CD8+ epitopes | |  |  |  |  |
| --- | --- | --- | --- | --- | --- |
| **Antigen** | **Residues** | **Sequence*** | **HLA restriction** | **Shared by** | **Residue change** |
| EBNA1 | 407-415 | HPVGEADYF | B53 | HKNPC1, GD1, GD2 | 411 E>A |
|  | 407-417 | HPVGEADYFEY | B35.01 | HKNPC1, GD1, GD2 | 411 E>A |
|  | 528-536 | IPQCRLTPL | B7 | HKNPC1, GD1, GD2 | 528 I>V |
| EBNA2 | 14-23 | YHLIVDTDSL | B38 | GD2, HKNPC1 | 23 L>R |
| EBNA3A | 458-466 | YPLHEQHGM | B35.01 | GD2, HKNPC1 | 459 P>T |
| EBNA3B | 399-408 | AVFDRKSDAK | A11 | GD2, HKNPC1 | 402 D>N |
|  | 416-424 | IVTDFSVIK | A11 | GD2, HKNPC1 | 424 K>N |
|  | 488-496 | AVLLHEESM | B35.01 | HKNPC1, GD1, GD2 | 488 A>T |
| EBNA3C | 213-222 | QNGALAINTF | B62 | HKNPC1, GD1, GD2 | 213 Q>H |
|  | 335-343 | KEHVIQNAF | B44.02 | HKNPC1, GD1, GD2 | 336 E>D |
|  | 343-351 | FRKAQIQGL | B27.05 | HKNPC1, GD1, GD2 | 348 I>L |
| LMP1 | 38-46 | FWLYIVMSD |  | HKNPC1, GD1, GD2 | 46 D>N |
|  | 72-82 | FRRDLLCPLGA | B40 | HKNPC1, GD1, GD2 | 82 A>G |
|  | 125-133 | YLLEMLWRL | A2 | HKNPC1, GD1, GD2 | 126 F>L |
| LMP2 | 141-154 | ASCFTASVSTVVTA |  | GD2, HKNPC1 | 153 T>S |
|  | 200-208 | IEDPPFNSL | B40.01 | GD2, HKNPC1 | 208 L>I |
|  | 240-250 | RRLTVCGGIMF | B27 | GD2, HKNPC1 | 248 I>M |
|  | 243-251 | TVCGGIMFL | A1 | GD2, HKNPC1 | 248 I>M |
|  | 249-262 | MFLACVLVLIVDAV |  | GD2, HKNPC1 | 254 V>L, 255 L>V |
|  | 340-350 | SSCSSCPLSKI | A11 | GD2, HKNPC1 | 350 I>V |
|  | 349-358 | ILLARLFLY | A29 | GD2, HKNPC1 | 350 I>V |
|  |  |  |  |  |  |
| CD4+ epitopes | |  |  |  |  |
| **Antigen** | **Residue** | **Sequence** | **HLA restriction** | **Shared by** | **Residue change** |
| EBNA1 | 403-417 | RPFFHPVGEADYFEY |  | HKNPC1, GD1, GD2 | 411 E>A |
|  | 429-448 | VPPGAIEQGPADDPGEGPST |  | HKNPC1, GD1, GD2 | 439 A>T |
|  | 434-458 | IEQGPTDDPGEGPSTGPRGQGDGGR | | HKNPC1, GD1, GD2 | 439 A>T |
|  | 509-528 | VYGGSKTSLYNLRRGTALAI | DR11 | HKNPC1, GD1, GD2 | 528 I>V |
|  | 515-528 | TSLYNLRRGTALAI | DR1 | HKNPC1, GD1, GD2 | 528 I>V |
|  | 518-530 | YNLRRGTALAIPQ | DP3 | HKNPC1, GD1, GD2 | 528 I>V |
|  | 519-533 | NLRRGRTALAIPQCRL |  | HKNPC1, GD1, GD2 | 528 I>V, 533 L>I |
|  | 527-541 | AIPQCRLTPLSRLPF | DR13 | HKNPC1, GD1, GD2 | 528 I>V, 533 L>I |
|  | 529-543 | PQCRLTPLSRLPFGM | DR14 | HKNPC1, GD1, GD2 | 533 L>I |
| EBNA2 | 276-295 | PRSTVFYNIPPMPLPPSQL | DR7,52a,52b,52c | GD2, HKNPC1 | 280 T>N |
|  | 280-290 | TVFYNIPPMPL | DQ2/DQ7 | GD2, HKNPC1 | 280 T>N |
| LMP1 | 11-30 | GPPRPPLGPPLSSSIGLALL | DR7 & DR9 | HKNPC1, GD1, GD2 | 13 R>P |
|  | 130-144 | LWRLGATIWQLLAFF |  | HKNPC1, GD1, GD2 | 144 F>I |
|  | 181-206 | LIWMYYHGPRHTDEHHHDDS | DR16 | HKNPC1, GD1, GD2 | 189 Q>P, 192 S>T |
|  | 206-225 | QATDDSSHESDSNSNEGRHH | DQ2 | HKNPC1, GD1, GD2 | 212 G>S |
|  | 211-236 | SSHESDSNSNEGRHHLLVSG | DQB1*0601 | HKNPC1, GD1, GD2 | 212 G>S |
|  | 212-226 | SGHESDSNSNEGRHHH |  | HKNPC1, GD1, GD2 | 212 G>S |
| LMP2 | 149-163 | STVVTATGLALSLLL |  | GD2, HKNPC1 | 153 T>S |
|  | 169-182 | SSYAAAQRKLLTPV |  | GD2, HKNPC1 | 169 S>N, 171 Y>S |
|  | 189-208 | VTFFAICLTWRIEDPPFNSI | DRB1*0901 | GD2, HKNPC1 | 208 L>I |
|  | 194-213 | ICLTWRIEDPPFNSILFALL | DRB1*1001 | GD2, HKNPC1 | 208 L>I |
|  | 385-398 | STEFIPNLFCMLLL |  | GD2, HKNPC1 | 391 N>H |

*Residues subject to changes are underlined
