# Supplementary material for: Genomic Sequencing and Comparative Analysis of Epstein-Barr Virus Genome Isolated from Primary Nasopharyngeal Carcinoma Biopsy
Source: PLoS One. 2012 May 10;7(5):e36939. doi: 10.1371/journal.pone.0036939 (PMC3349645; doi:10.1371/journal.pone.0036939)
Supplement: Table S4 — Primers for EBV enrichment in next-generation sequencing. (DOCX) [file pone.0036939.s004.docx]

**Table S4. Primers for EBV enrichment in next-generation sequencing.**

| **Primer Pairs** | **Product size** | **Product coordinates** |
| --- | --- | --- |
| TTCTGGTGATGCTTGTGCTC | 2076 | 540-2616 |
| TGCTGGCGTCTCATAAACAG |  |  |
| CTGTTTATGAGACGCCAGCA | 4764 | 2597-7361 |
| TTTTCGCTGCTTGTCCTTTT |  |  |
| CCTGTGTGACCCCTCACTTT | 4567 | 5545-10112 |
| TCCTTTTTCCTGCAGCTTGT |  |  |
| AAAAGGACAAGCAGCGAAAA | 4307 | 7342-11649 |
| GTGCAGGAGGCTGTTTCTTC |  |  |
| TTATGGTTCAGTGCGTCGAG | 4007 | 10971-14978 |
| GAACTGAGGAGGGCATGAAG |  |  |
| ATGCCTACATTCTATCTTGCGTTAC | 1439 | 36216-37655 |
| TTACTGGATGGAGGGGCGAGGTCTT |  |  |
| AGGGATGCCTGGACACAAGA | 1412 | 36522-37934 |
| AACATGGACTGGGAGTGGAG |  |  |
| CTAGAGGTCCGCGAGATTTG | 1251 | 40696-41947 |
| AGAAGGCAAGCGAAAATTGA |  |  |
| GCAGGCAGTACGAGATGTCA | 2239 | 41700-43939 |
| TCCCTTCACATCCCAGAGAC |  |  |
| CGACATTGACAGCCTTCTCA | 4312 | 43795-48107 |
| AAACACGAATGCCAAGAACC |  |  |
| TGCTCCTGATGTTTCTGAGGTGGA | 1776 | 47586-49362 |
| AGGTAACTTCTTTGAGCCTCCCGA |  |  |
| TTGCTCCATCTGTCAGCAAC | 1759 | 49088-50847 |
| CACAAGCCTCCTCTCAGGAC |  |  |
| GGTGACCACTGAGGGAGTGT | 1789 | 50045-51834 |
| CTTTCGAGCCAGAGATGTCC |  |  |
| CCGAAATAGGGCCTTGCCATCAAT | 1046 | 51199-52245 |
| ATTTCAGGACTACCTGCGCGACTT |  |  |
| GGACATCTCTGGCTCGAAAG | 4689 | 51815-56504 |
| AGGAGGAGAACCCGAGGATA |  |  |
| TCAGGAGGTCGTCAAAATCC | 1720 | 56125-57845 |
| AGTAATCCCCATCCCTCACC |  |  |
| TCCAGGCTGTTGGAGAACACTTCA | 1172 | 57133-58305 |
| TTTCACATCCGACTCATTCCCTGC |  |  |
| GGTGAGGGATGGGGATTACT | 2035 | 57826-59861 |
| ATCACAGTCACCCCCAGAAG |  |  |
| CCAGTCGCCGTTACTCATCT | 1768 | 59222-60990 |
| GCTCATATACGCCACCGTCT |  |  |
| CAGACGGTGGCGTATATGAG | 4019 | 60970-64989 |
| CAAAGAGCCCCGTAAAGATG |  |  |
| AACAGGCGGGCGAATGTGTAAT | 1272 | 60125-61397 |
| ACCTTTCATCCGAACTCCTCAGGT |  |  |
| GCCTCTATGTCGCTCTGACC | 4771 | 63737-68508 |
| CGGAGGCGTGGTTAAATAAA |  |  |
| GCGAGCCATAAAGCAGTTTC | 4647 | 67334-71981 |
| TCTCCCGAACTAGCAGCATT |  |  |
| CTCGCGTGTTAGGAAGGAAG | 5649 | 70814-76463 |
| AGGCAAAGCTGGTCAAAGAA |  |  |
| AGAAGCGCCGGTACTTGTTAAGGA | 1495 | 75554-77049 |
| TTGATTCTCGTGGTCGTGTTCCCT |  |  |
| GCCTTCTTTGACCAGCTTTG | 5215 | 76441-81656 |
| GACGGGTTCTACTGGCATGT |  |  |
| GGTGAAACGCGAGAAGAAAG | 4797 | 81084-85881 |
| TTTAGCAGTTCCTCCGCACT |  |  |
| CCCCATCAGACACCTCAAGT | 2138 | 85093-87231 |
| CACCTCCCGTTGCTAACATT |  |  |
| AGTGCGGAGGAACTGCTAAA | 2917 | 85861-88778 |
| TGCAGAGGATGAGACCAGTG |  |  |
| CCCACCACGTCTTCAACTTT | 2088 | 88730-90818 |
| CCATACCAGGTGCCTTTTGT |  |  |
| TCCAAGGTGACCCCTGTTAG | 4859 | 89141-94000 |
| TGATGCAGAGTCGCCTAATG |  |  |
| ACTCCCGGCTGTAAATTCCT | 4923 | 92774-97697 |
| TGGCCAGAAATACACCAACA |  |  |
| GTGATGAGGACGAGGATGGT | 4100 | 95485-99585 |
| TCGTGGATGCCCTAAAGAAC |  |  |
| CCCATGTTGTCACGTCACTC | 5281 | 97747-103028 |
| CACCGTGTTGGAGACCTTTT |  |  |
| ACAGACCATCTACGCCAACC | 5392 | 102044-107436 |
| CCACCACAAGAAGGTGTCCT |  |  |
| TACGGGGCACTTAACCTGAC | 4005 | 107002-111007 |
| TGACGGAGCTGTATCACGAG |  |  |
| GATGTTGCTGGGGCTAATGT | 4358 | 110724-115082 |
| AGAGAGGGAGTTTCGCTTCC |  |  |
| GGCACCATAGCATGTCACAC | 4571 | 113938-118509 |
| AGTCCCAACAACTTCCAACG |  |  |
| AACACCATCCAGCTCTCCTTCGAT | 1526 | 117867-119393 |
| ATGGGTGTCCGACCAATCCATTCT |  |  |
| CGTTGGAAGTTGTTGGGACT | 4144 | 118490-122634 |
| CATTTTACCAGGGACGAGGA |  |  |
| ATGCACCTCAAAGGTTACCG | 4589 | 121912-126501 |
| TTGCAAACTCGCATCTTCAC |  |  |
| CCCGTTCACCAAAACAGTCT | 4445 | 122398-126843 |
| AACCAGGACACGTTGAGACC |  |  |
| GGTCTCAACGTGTCCTGGTT | 4287 | 126824-131111 |
| GTGAAGGTATGTGCCGGTCT |  |  |
| ACCTCCCATAGCAACACCAG | 4195 | 130704-134899 |
| CCCGTGCGATGAGTTTATTT |  |  |
| CCTGAGAACGCTCCAGGTAG | 4136 | 133032-137168 |
| CCTGGTGAGAAGTTGGTGGT |  |  |
| CCAGACATACCCCAAACCAC | 4360 | 136001-140361 |
| CTCCAGAGGGCAGACGTTAG |  |  |
| GCCCGTTGGGTTACATTAAGGTGT | 1437 | 143534-144971 |
| CATGCAGTGGTGTCAGACAGGAAA |  |  |
| TTTGGGATGCATCACTTTGA | 3279 | 144203-147482 |
| CCTCAAAGGTGTGGTCGTTT |  |  |
| CTTTGGGTTCCATTGTGTGCCCTT | 2838 | 147061-149899 |
| ACCTGGTACATTGTGCCCATCAGA |  |  |
| TCGTGGCTCGTACAGACGATTGTT | 3411 | 147344-150755 |
| TTTGCGCCTTCTCCTGGTTTATGC |  |  |
| CCCACACCTTCACTCCTTGT | 4002 | 151117-155119 |
| CAGAGCCAGGCACATCTACA |  |  |
| TAGTAGCGGGCAACGAGAGA | 4030 | 151842-155872 |
| CGTGTGTGTGAACGTGTTTG |  |  |
| ACGCCATACCCAAGTGAGTC | 5403 | 152284-157687 |
| TCAAGAACCTGACGGAGCTT |  |  |
| TGGAAGAAGGCGTAGAGCAT | 4181 | 155195-159376 |
| CTTGTTTACCCAGACCCTGA |  |  |
| ACGCCGAGTCATCTCTCATTTGGA | 1080 | 158854-159934 |
| GCAAGGCTGACTCACCTGTTTGA |  |  |
| GCTCAGGGTCTGGGTAAACA | 2368 | 159355-161723 |
| CGTGACTACCCCCACGTACT |  |  |
| AGGTTGCACACCACATCAAA | 3871 | 161261-165132 |
| GACTCGCTCACCCAAGAAAG |  |  |
| GTGCAGAGCCTTGACATTGA | 4183 | 164101-168284 |
| TGAACACCACCACGATGACT |  |  |
| CACGGGGTTTATGTTTCTGG | 4002 | 165676-169678 |
| CCCCCTCCACTTTTTCCA |  |  |

*Coordinates of NC007605
