# Supplementary material for: Genomic Sequencing and Comparative Analysis of Epstein-Barr Virus Genome Isolated from Primary Nasopharyngeal Carcinoma Biopsy
Source: PLoS One. 2012 May 10;7(5):e36939. doi: 10.1371/journal.pone.0036939 (PMC3349645; doi:10.1371/journal.pone.0036939)
Supplement: Table S5 — Primers for validation by Sanger sequencing. (DOCX) [file pone.0036939.s005.docx]

**Table S5. Primers for validation by Sanger sequencing.**

| **Primer Pairs** | **Product Size** | **Product Coordinates** |
| --- | --- | --- |
| TCGTCTTGCTCTATTCACCCT | 611 | 6-617 |
| ACATCATGCCGCCACAAACAGT |  |  |
| TGAATCCAGTATGCCTGCCTGT | 557 | 60-617 |
| ACATCATGCCGCCACAAACAGT |  |  |
| ACACGTGTGACATTGCTTGCCT | 525 | 796-1320 |
| GAGTATGCCAGCGACAATCAGT |  |  |
| AAGCAGCTCACCTATGGTCACTCA | 440 | 5646-6085 |
| ATAGTTTCGAGTGGTGAGGAGGGA |  |  |
| AGCCGTTGCCCTAGTGGTTTC | 1464 | 6961-8424 |
| TGGTTGTCTCTAACACCCCCGA |  |  |
| AAGCAGCGAAAATTCACGCCC | 894 | 7350-8242 |
| TCGCTAACCACTGCGGTCAA |  |  |
| AGGTGGAGCAACGTCTAAAGTGGT | 515 | 11134-11648 |
| TGCAGGAGGCTGTTTCTTCAGT |  |  |
| TGGTCCTGCAGCTATTTCTGGT | 457 | 14359-14660 |
| TTACCACCTCCTCTTCTTGCTGGA |  |  |
| TCTTTGGGCCCTCAAGTCCA | 533 | 13191-13703 |
| AGGCCCAAGGGTCACATAAAGT |  |  |
| TTCTGCTAAGCCCAACACTCCA | 706 | 14640-15346 |
| TGCTCGTTACCAGAGAGAATGG |  |  |
| ACCTCTCCAGGGCTCACATAAA | 3407 | 33277-36684 |
| TGTGGGCCTTGTGGCAACATAA |  |  |
| TATGTTGCCACAAGGCCCACAA | 988 | 36665-37652 |
| ACTGGGTCCCACCACATAATCT |  |  |
| AGATTATGTGGTGGGACCCAGT | 658 | 37631-38289 |
| TAGTGCAGTGTCCCTGCTGC |  |  |
| ACTCCTATGCATTTCCTGCCCT | 1889 | 37938-39827 |
| ACCCACCTGGTGACACACCTTAAT |  |  |
| TTGAGTCTCTCGAGGTCTGCGAT | 567 | 49806-50372 |
| TGTCAGGAGCAGACAGCAACAA |  |  |
| GAGCTATGCCTCCCGTCGAC | 378 | 50899-51276 |
| GGGACGTGACAGTTACCCCG |  |  |
| GAAAGAGTCGTGGTGGTGGGG | 1213 | 57630-58842 |
| CGGCGGAGATCTTTGGCCTC |  |  |
| AGTGGTGGAGGACAGAGGGA | 609 | 57923-58531 |
| CCCGAGGCCTTTGAGACAGAG |  |  |
| TCATCTTCGGTGGTGGGACG | 526 | 59236-59760 |
| CCTCACCCGTGGGACAAACG |  |  |
| TATTTGCAAAGGGAGGCGAGGA | 1070 | 57734-58803 |
| TCGCAAACTCGGCAGTTGTTCA |  |  |
| GGCGAACTGGTGGACACATGA | 802 | 77707-78508 |
| ACGTGCCTACCAACCTCACC |  |  |
| AAATCGTGACACTGGACGTGGT | 550 | 79044-79576 |
| TTGGTGCAGGAGAATTGGCTCT |  |  |
| AGCTCAGGGAATGGCATACACA | 978 | 81398-82375 |
| ATTGGCGGCTGATGGAGGAAAT |  |  |
| AAGCCATGGTTAAGAGCGCA | 606 | 84073-84678 |
| ACACCCTGCATGGATTCTTCGT |  |  |
| AATGCCACTGCGACCTATCCCTAT | 618 | 85017-85634 |
| CCCATAACCTGTATGGGCTGCAAA |  |  |
| AGCGTGTGTTGTGTACGACGAT | 605 | 87661-88263 |
| AACTTTGTATGGGCTGGGCCTT |  |  |
| ATACAGTCACCCGACAATGAGC | 311 | 86115-86423 |
| GCATCCCTGATATTCCTTGCGA |  |  |
| CTGCTTCAACAGGAGGCGCA | 495 | 90001-90494 |
| CAGCAGCAGTGGTGTTGGCT |  |  |
| CATGTCTGACGAGGGGCCAG | 1133 | 95661-96791 |
| CACCACGTCCACGACCTCTC |  |  |
| AAGTTGCATTGGCTGCAAAGGG | 947 | 95892-96838 |
| ACCCGGATGATGATGACTGACT |  |  |
| TTGCATTGGCTGCAAAGGGG | 614 | 96177-96791 |
| CACCACGTCCACGACCTCTC |  |  |
| TCAGTCATCATCATCCGGGTCT | 523 | 96819-97341 |
| ACAGACAATGGACTCCCTTAGTGG |  |  |
| TTTTCAAAAGCATGCGCCAGGG | 720 | 107822-108540 |
| GGGAAACCAACGAGGGGCTC |  |  |
| TTTTCAAAAGCATGCGCCAGGG | 449 | 107822-108271 |
| GGACCCGATCAGAGCACCATG |  |  |
| CAGGCCTAAATACGTGGTGGGG | 1016 | 138271-139286 |
| ACCTGGTGCCTACGTGATGTG |  |  |
| ACGGATAAGGACCGCGGAGT | 472 | 138814-139286 |
| ACCTGGTGCCTACGTGATGTG |  |  |
| ACGGGAGAATTTGGGCTCGTTT | 505 | 139620-140125 |
| TTGCAGGTGTGAAATGCTTGGC |  |  |
| GCCAAGCATTTCACACCTGCAA | 3999 | 140104-144102 |
| AAGGTGACAAGTCAGGAAGGCA |  |  |
| GGCTAGAGGCCCGCGAGATT | 1060 | 143613-144673 |
| GCGGGCGCCCATTAGAATCT |  |  |
| GGCTAGAGGCCCGCGAGATT | 1978 | 143613-145591 |
| GTGGTGACATTTGGGACCGGA |  |  |
| TCCGGTCCCAAATGTCACCAC | 688 | 145571-146258 |
| CAGCTCAGTGACACGCCCAAT |  |  |
| GTGGCAGCTGTTGTTTGTACTGG | 863 | 146942-147804 |
| CACCGGAAATTGGCACCGCA |  |  |
| TTTTTATGGGCGAGTGGGCG | 867 | 150527-151393 |
| GCCTGCGTTGCCTAAATCCT |  |  |
| CCTCCTTGGGTGGAACGACG | 770 | 150659-151428 |
| GGTGCACGCTCCCATGTTTG |  |  |
| TTTTCTGCATTCGCCCTTGCG | 441 | 152749-153189 |
| AAACGAGAGAAGAGAGCGGCG |  |  |
| TCAGCACCATGTTCTGCCTCTT | 507 | 157382-157889 |
| TTCGTGACCAACACAACCGT |  |  |
| GGAGAGTCAGTCAGGCAAGCCT | 610 | 167648-168257 |
| GCACCCTCAACAAGCTACCG |  |  |
| AAGGTGTCTGCCAATTCTCGCA | 1142 | 167828-168647 |
| TGGAATTTGCACGGACAGGCAT |  |  |
| TTCAGAATGAGGTGGCGGATTC | 588 | 169550-170138 |
| TTTGTCAGGGTTGCCTGTGTCA |  |  |
| ACGCCGTTGGAGGGTAGAATGA | 534 | 171640-250 |
| TGTAACCGGTGTCAGCAGTTTCCT |  |  |

*Coordinates of NC007605
